# Nef gene evolution from a single transmitted strain in acute SIV infection

**DOI:** 10.1186/1742-4690-6-57

**Published:** 2009-06-08

**Authors:** Benjamin N Bimber, Pauline Chugh, Elena E Giorgi, Baek Kim, Anthony L Almudevar, Stephen Dewhurst, David H O'Connor, Ha Youn Lee

**Affiliations:** 1 Wisconsin National Primate Research Center and Department of Pathology and Laboratory Medicine, University of Wisconsin–Madison, Madison, Wisconsin 53706, USA; 2 Departments of Microbiology and Immunology, University of Rochester Medical Center, Rochester, New York 14642, USA; 3 Theoretical Biology and Biophysics, Los Alamos National Laboratory, Los Alamos, New Mexico 87545, USA; 4 Mathematics and Statistics, University of Massachusetts, Amherst, Massachusetts 01002, USA; 5 Biostatistics and Computational Biology, University of Rochester Medical Center, Rochester, New York 14642, USA

## Abstract

**Background:**

The acute phase of immunodeficiency virus infection plays a crucial role in determining steady-state virus load and subsequent progression of disease in both humans and nonhuman primates. The acute period is also the time when vaccine-mediated effects on host immunity are likely to exert their major effects on virus infection. Recently we developed a Monte-Carlo (MC) simulation with mathematical analysis of viral evolution during primary HIV-1 infection that enables classification of new HIV-1 infections originating from multiple versus single transmitted viral strains and the estimation of time elapsed following infection.

**Results:**

A total of 322 SIV *nef *SIV sequences, collected during the first 3 weeks following experimental infection of two rhesus macaques with the SIVmac239 clone, were analyzed and found to display a comparable level of genetic diversity, 0.015% to 0.052%, with that of *env *sequences from acute HIV-1 infection, 0.005% to 0.127%. We confirmed that the acute HIV-1 infection model correctly identified the experimental SIV infections in rhesus macaques as "homogenous" infections, initiated by a single founder strain. The consensus sequence of the sampled strains corresponded to the transmitted sequence as the model predicted. However, measured sequential decrease in diversity at day 7, 11, and 18 post infection violated the model assumption, neutral evolution without any selection.

**Conclusion:**

While *nef *gene evolution over the first 3 weeks of SIV infection originating from a single transmitted strain showed a comparable rate of sequence evolution to that observed during acute HIV-1 infection, a purifying selection for the founder *nef *gene was observed during the early phase of experimental infection of a nonhuman primate.

## Background

Genetic evolution in the primary phase of HIV-1 infection has been characterized by single genome amplification and nested polymerase chain reaction (PCR) of HIV-1 genes in parallel with mathematical/computational modeling [1-3]. Major goals of such analyses include the characterization of the transmitted strains, estimating the timing of infection based on the level of sequence diversity, and distinguishing between single virus strain/variant infections (referred to hereafter as "homogenous" infection) versus two or more virus strains/variants infections (referred to hereafter as "heterogenous" infection). Heterogeneous infection is associated with faster sequence diversification and accelerated disease progression due to the rapid emergence of virus variants with enhanced replicative fitness [4-7].

To quantitatively assess whether HIV-1 infections were initiated by single or multiple viral strains, we recently developed a mathematical model and Monte-Carlo (MC) simulation model of HIV-1 evolution early in infection and applied this to the analysis of 102 individuals with acute HIV-1 infection [2]. Further, in cases of single strain (homogeneous) infections, the model provided a theoretical basis for identifying early founder (possibly transmitted) *env *genes.

In this study, we tested the validity of our primary HIV-1 infection model using a non-human primate (NHP) model for HIV-1/AIDS. This model has played a key role in the development of candidate HIV-1 vaccines, and provided critical insights into disease pathogenesis [8-10]. Studies in the macaque/simian immunodeficiency virus (SIV) model have contributed to our understanding of the close association between the extent of virus replication during the acute phase of infection and the subsequent virus set point and disease course [11] as reported in HIV-1 infections [12-14]. Genetic evolution during SIV infection has been well documented in comparison with the evolution of HIV-1 population [15-18].

We examined evolution of the viral *nef *genes from a single transmitted strain. Nef, a small accessory protein, was selected because the virus can tolerate significant variability in the nef protein, as evidenced by high levels of polymorphism longitudinally throughout infection and at the population level [19-22]. We sequenced full-length nef genes longitudinally during the very early phase of SIV infection using the method of single genome amplification (SGA). The SGA method more accurately represents HIV-1 quasispecies when compared to conventional PCR amplification [1,23,24]. We showed that our sequence evolution model correctly classified the experimental SIV infections as homogeneous infections. As predicted by the model, the consensus sequence of the sampled strains from these homogeneous infections corresponded to the transmitted sequence. However, our systematic evaluation showed that a sequential decrease of the diversity within the first 3 weeks of infection was associated with a purifying selection for the transmitted sequence (and was not a consequence of the limited sample size in our analysis).

## Results

### Longitudinal nucleotide and amino acid mutations

**Figure 1:**
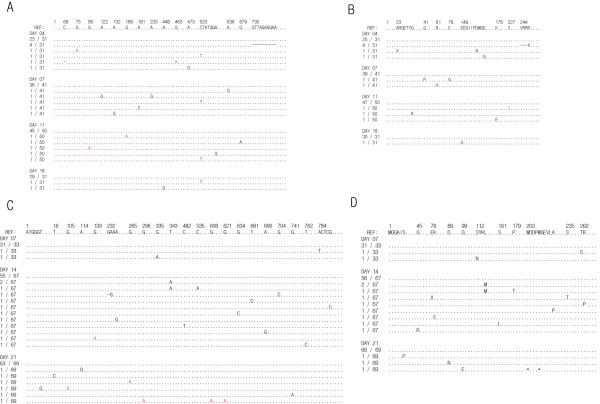
**Nucleotide and amino acid base substitutions within 3 weeks post SIV infection**. Longitudinal nucleotide (A) and amino acid (B) base substitutions from the founder nef gene/Nef protein of sequence samples taken at day 4, 7, 11 and 18 post-infection from animal r00065, which was infected intravenously with SIVmac239. C and D display base substitutions in reference to the founder sequence from the samples taken at day 7, 14, and 21 post-infection from animal r98018, which was infected by intrarectal inoculation with SIVmac239. Numbers in the left column in each figure represent the number of a specific sequence out of total sampled sequences at a given day post infection. Each clone was obtained via the method of single genome amplification.

We visualized longitudinal sequence evolution, nucleotide and amino acid point mutations in reference to the founder nef gene/Nef protein in Figure 1. From a total of 322 *nef *sequences sampled from the two animals, we observed 41 nucleotide base substitutions (excluding gaps) from the infecting *nef *sequence of SIVmac239, within the first 21 days following virus infection; out of these 41 mutations, 10 were determined to be G-to-A hypermutation patterns with APOBEC signatures (red characters in Figure 1) [25]. However, none of these APOBEC signatures were statistically significant (p > 0.05 from a Fisher exact test, Hypermut tool ). As we predicted in our model [2], the group sequences identical to the consensus sequence indeed corresponded to the transmitted *nef *sequence. Limited base substitutions observed in all nef genes were sparse and did not align with each other – as we have seen in *env *genes sampled from HIV-1 acute subjects classified as having homogeneous infection [2]. Out of 41 total mutations, 16 mutations were synonymous and the rest were non-synonymous base substitutions.

**Figure 2:**
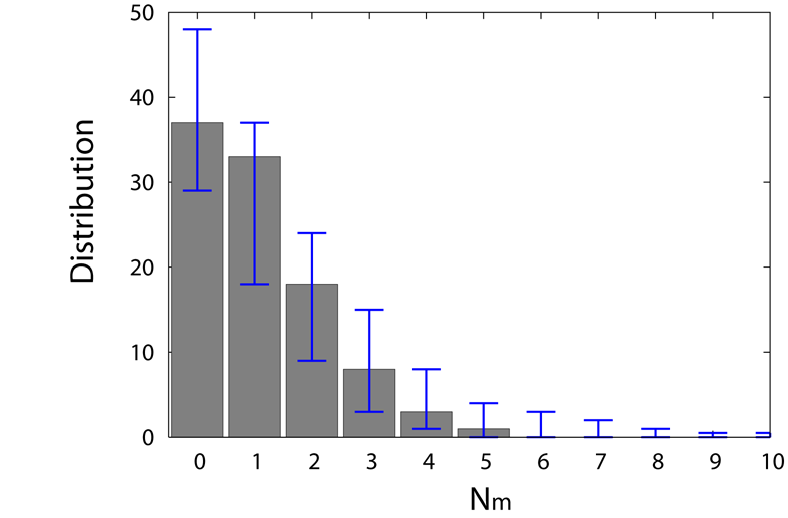
**Histogram of the observed number of mutant sequences sampled at more than one time point, *N*_*m*_**. At day 6, 12, 18, and 24 post infection, 30 nef sequences were sampled. The observed number of mutant sequences which were present at more than one time point was counted from the total of 120 sequences sampled sequentially over 4 time points. For example, *N*_*m *_= 0 denotes that no mutant sequence from the founder gene appeared at more than one time point. The histogram of *N*_*m *_with 95% CIs was constructed by repeating 10^2 ^asynchronous MC infection simulations. While the founder *nef *gene remains as the majority of the sampled sequences, loss of mutant sequences in the serial samples was frequently observed.

Figure 1 shows that all the mutant *nef *genes except one were not sampled again in the next time point, while the transmitted *nef *gene was conserved in sequential samples from both animals. A single mutation fixed in the sequence population from animal r00065, C-to-T at position 520, was synonymous one. We examined whether loss of mutant sequences in the sequential samples could be reproduced in the MC simulation. We sampled 30 sequences at days 6, 12, 18, and 24 post infection in the asynchronous infection MC simulation, and then counted the number of mutant sequences that remained at more than one time point, by repeating 10^2 ^simulations. Figure 2 shows the histogram of the observed number of mutant sequences sampled in any of the sequential time points, *N*_*m*_. The 95% confidence intervals were calculated by repeating 10^2 ^of 10^2 ^MC runs. The simulation confirmed that loss of mutant sequences is frequent. While the transmitted, founder *nef *gene remains as the majority of the sampled sequences throughout the early infection period, the mutant sequences are not fixed in the population due to i) only a finite number of sequences are sampled in an exponentially growing population and ii) more mutations to the mutant genes are accumulated by further reverse transcription events.

### Dynamics of divergence, diversity, variance, maximum HD, and sequence identity

Viral diversification in early infection can be probed with several quantities based on Hamming distances among the sampled sequences. Here Hamming distance denotes the number of bases at which any two sequences differ. We measured the kinetics of divergence, diversity, variance, maximum Hamming distance (HD), and sequence identity in the two experimentally infected macaques (Table 1). Divergence is defined as average Hamming distance per site from the transmitted *nef *gene. Diversity is defined as average intersequence Hamming distance per site, variance as variance of intersequence per base Hamming distance distribution, maximum HD as measured maximum Hamming distance between all sequence pairs, and sequence identity as the proportion of identical sequences to the transmitted strain.

**Table 1:** Animal Information and analysis using the acute HIV-1 infection model.

Animal Index -sample date	viral load (copies/ml)	Number of Sampled Sequences	Divergence	Diversity	Variance	Max. HD	Sequence Identity	Estimated days post infection (95% CIs)	*χ*^2^goodness of fit P value
r00065-day4	18,600	31	0.016%	0.033%	0.027%	2	87.1%	14 [4–35]	0.79

r00065-day7	1,660,000	41	0.018%	0.037%	0.043%	3	87.8%	16 [6–34]	0.52

r00065-day11	90,800,000	50	0.013%	0.025%	0.022%	2	90.0%	11 [4–25]	0.82

r00065-day18	39,750,000	31	0.0081%	0.016%	0.015%	2	93.5%	7 [1–25]	0.93

r98018-day7	20,000	33	0.0077%	0.015%	0.014%	2	93.9%	7 [1–23]	0.93

r98018-day14	12,380,625	67	0.026%	0.052%	0.055%	4	82.1%	22 [12–37]	0.70

r98018-day21	1,391,000	69	0.016%	0.033%	0.057%	5	91.3%	14 [7–27]	0.86

Animal information including time of sampling, viral load, and number of nef sequences obtained. For each sample, we calculate divergence, diversity, variance, maximum HD, and sequence identity. Estimated days since infection with 95% confidence intervals and p-values were calculated via Maximum Likelihood method to fit a Poisson distribution to Hamming distance distribution from the founder strain and the goodness of fit through a Chi-Square statistic.

**Figure 3:**
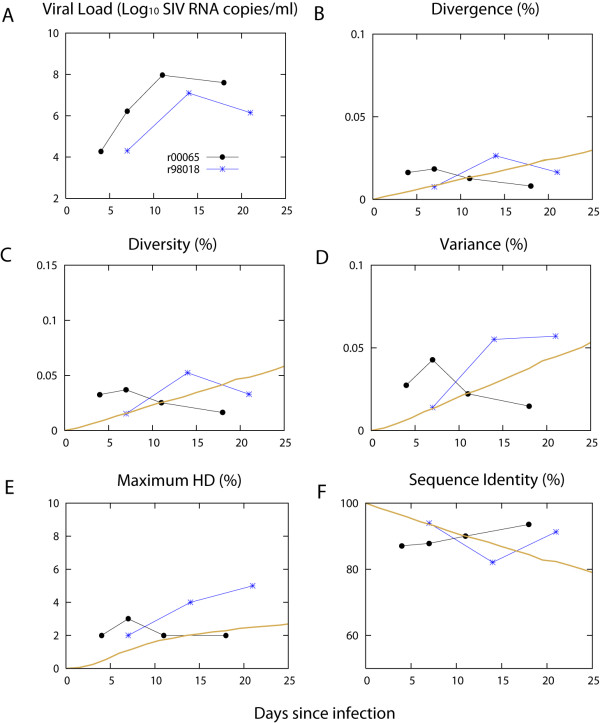
**Viral load kinetics and the dynamics of divergence, diversity, variance, maximum HD, and sequence identity from homogeneous SIV infection**. A. Viral load kinetics of animal r00065 (r65, black) and animal r98018 (r98, red). Animal r00065, which was infected by intravenous injection, displays a greater level of viral replication in comparison with animal r98018 which was infected by intrarectal inoculation. Dynamics of divergence (B), diversity (C), variance (D), maximum HD (E), and sequence identity (F) of nef sequences from animals r00065 (black) and r98018 (red). Each average value of simulated quantity from 10^3 ^simulations is represented with a brown line [2]. We sampled 31 sequences at a given time point in each run.

Figure 3 displays the kinetics of these quantities compared to the viral load dynamics for animal r00065 and animal r98018. Each measurement was in the range of the prediction made by our acute HIV-1 sequence evolution model, however, the dynamics of each quantity from the two serial samples was not consistent with that from the model prediction. For instance, the average HD from the founder *nef *gene, divergence, decreases from 0.018% to 0.0081% over a time interval of 11 days for animal r00065, which is opposite to the trend predicted by the model. Also the proportion of identical sequences to the transmitted one was serially elevated from day 7 to day 18, suggesting either a purifying selection back to the founder strain during the early stage of infection or stochastic fluctuations due to the limited sample size.

**Figure 4:**
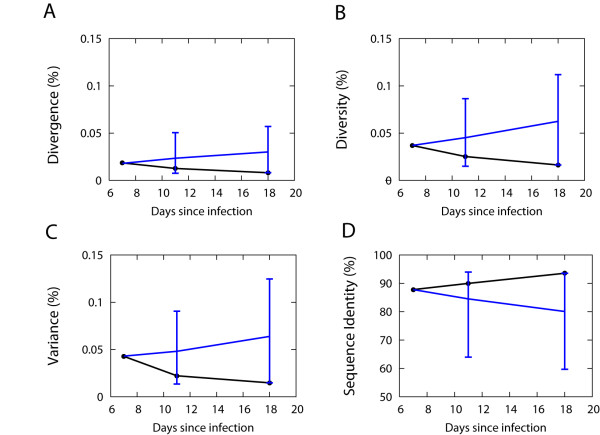
**Predicted divergence, diversity, variance, and sequence identity from a simulation performed by starting with 41 sampled *nef *sequences obtained at day 7 from animal r00065**. 50 sequences at day 11 and 31 sequences at day 18 were sampled by starting a simulation with the 41 sampled *nef *genes that were obtained at day 7 from animal r00065. The sampling time points were chosen to reflect those used in our initial simulation (i.e., day 11 corresponds to day 4 following the "initial" infection in this simulation, and day 18 corresponds to day 11 following the "initial" infection. The measured divergence at day 18, 0.0081%, from animal r00065 is located outside of the 95% confidence intervals of the predicted divergence at day 18, [0.00815%, 0.057%].

To address whether the acute stage sequence evolution in animal r00065 indeed shows a purifying selection back to the founder strain, we performed a MC simulation by starting with 41 *nef *sequences identical to those sampled at day 7 from animal r00065. Then we sampled 50 sequences at day 11 (4 days since the "starting" day 7) and 31 sequences at day 18 (11 days since the "starting" day 7) to replicate the experimental sampling from animal r00065. Figure 4 shows each measure of divergence, diversity, variance, and sequence identity with 95% confidence intervals from 1000 MC runs. The measured divergence at day 18, 0.0081%, from animal r00065 is located outside of the 95% confidence intervals of the predicted divergence at day 18, [0.00815%, 0.057%], denoting a violation of the model assumption, neutral evolution without selection. We conclude that the serial decrease in divergence observed in animal r00065 is reflective of a purifying selection rather than a stochastic effect from the finite size of sampling.

The maximum HD of r98018 at day 21 is 5 due to the presence of a strain with 3 base substitutions from the founder strain. All three of these mutations are G to A hypermutation with APOBEC3G/F signatures [25-27], although the signatures were not found to be statistically significant (p > 0.05 from a Fisher exact test, Hypermut tool ). Nonetheless, we tentatively attribute the deviation from the prediction generated by our model to these putative APOBEC3G/F signatures. The rate of virus sequence evolution in animal r00065 was slower than in animal r98018 – even though the virus replication rate (virus load) in animal r00065 was higher than that for animal r98018.

### Single Variant (Homogeneous) Infection with Neutral Evolution

**Figure 5:**
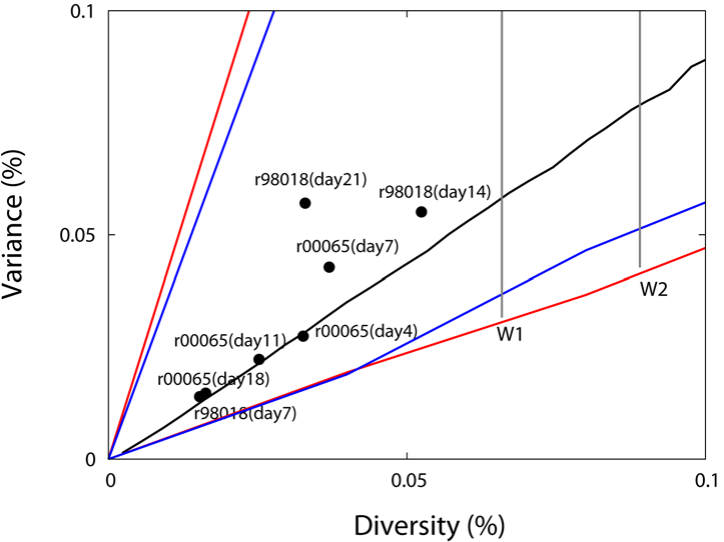
**Classification diagram for homogeneous infection**. The diversity and the variance of the sampled sequences from animals with homogeneous infection (i.e. infections with a single founder strain without any selection pressure or recombination) are expected to be located within the conical region. Here, the red (blue) conical region represents the 95% CIs from 5 × 10^3 ^runs where 20 (60) sequences were sampled at each time point. The black diagonal line denotes the average relationship between diversity and variance. The grey vertical line denotes the upper limit of the 95% CIs of simulated diversity at each time point. All of the sequence sets sampled from the two primates within 3 weeks since infection were successfully classified as homogeneous infections; measured diversity and variance are located within the red and blue conical regions and the diversity is less than the upper limit of the 95% CIs of diversity at week 1 from the homogeneous infection simulations.

Our MC simulation and mathematical calculation is based on the premise that the SIV sequence population diversifies through random base substitutions without any selection or recombination during the first 2–3 weeks of infection, prior to initiation of the host nef-specific immune response that could select viral escape variant. Based on this assumption, the Hamming distance distribution can be approximated as a Poission distribution which is characterized as mean (diversity) equals variance [2,28]. The equality will not be exact due to stochastic effects and sample size dependency. However, we can use the simulation output to capture these effects, and construct a conical region delimited by 95% CIs over mean and variance within which values from a sample from homogeneous infection should lie (Figure 5). If we sample more sequences, the area of the cone decreases. The two conditions for the single variant homogeneous infection without any selection or recombination are: i) measured diversity and variance of the sequence sample should be located inside the cone, between the upper and lower limits of the 95% CIs, and ii) diversity should be less than the upper limit of the 95% CIs of simulated diversity at a given time point (grey lines in Figure 5). Here the cone diagram in Figure 5 was constructed by measuring diversity and variance for 20 (red) or 60 (blue) *nef *genes at each time point of each MC run. We performed 5000 MC runs. All the homogeneous 7 sequence samples from the two animals satisfy the above two conditions, as Figure 5 depicts. Our model successfully classified the virus sequence pattern in the two animals as being derived from a "homogeneous" infection as opposed to a "heterogeneous" infection with two or more strains.

### Estimating Days since Infection: Poisson Fit

**Figure 6:**
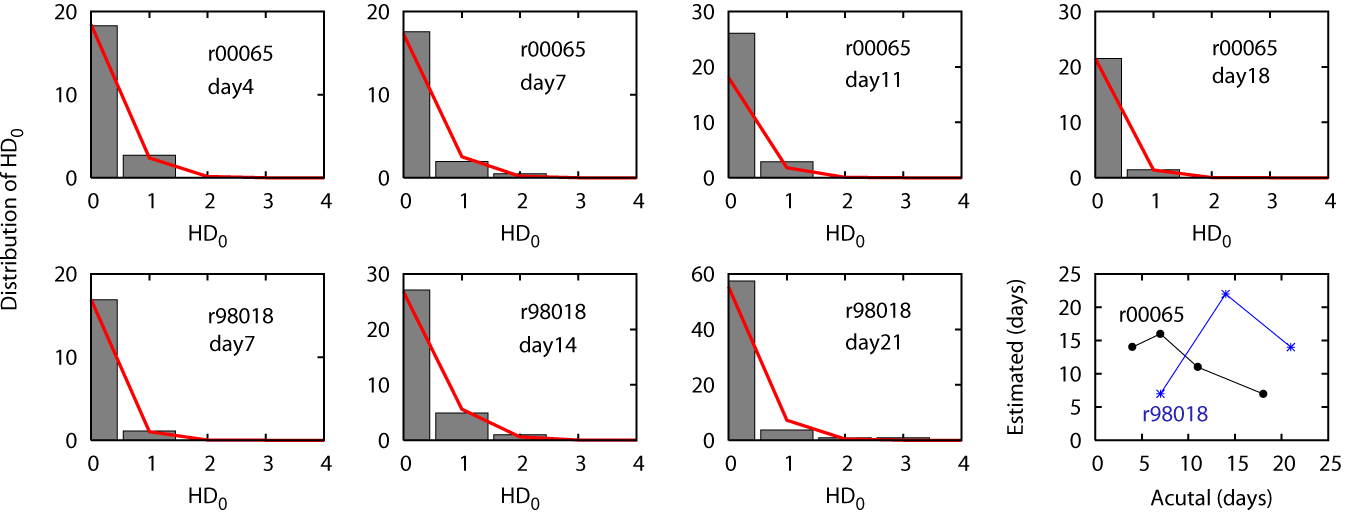
**Estimation of days since infection based on Hamming distance distribution**. The Hamming distance (*HD*_0_) distribution (multiplied by the number of sampled sequences) from the founder *nef *strain, SIVmac239, is shown for each sequence sample from each animal (black boxes) with the best fitting Poisson distribution (red lines). The goodness-of-fit p value of each fit is listed in Table 1. The bottom right corner panel shows a comparison between actual days post infection and the estimated days since infection based on *HD*_0 _distribution for animals r00065 (black) and r00098 (blue). The correlation coefficient between the actual and estimated dates post-infection for r00065 is -0.91 and for r98018 is 0.47.

For each sequence data set, which was sampled from each animal at a time point following infection, we constructed the distribution of Hamming distances from the founder strain, *HD*_0 _(Figure 6). The distribution of Hamming distances from the founder strain, *HD*_0_, was calculated as a weighted sum of Binomial distributions in the asynchronous infection mathematical model. The weighted sum of Binomial was approximated as a Poisson distribution,

(1)

with the mean of

(2)

where . Here *t *is days post infection, *ε *is the HIV-1 single replication cycle error rate per base, *N*_*B *_is the number of bases of sampled genes, and *R*_0 _is the basic reproductive ratio.

We used a Maximum Likelihood method to fit a Poisson distribution to the observed data, and then assessed the goodness of fit through a Chi-Square statistic. Table 1 summarizes the estimated days since infection obtained from the Poisson fit using the relationship between mean of Poisson distribution, *λ*_0 _and days post infection, *t *in Eq. (2), along with 95% CIs obtained by bootstrapping the *HD*_0 _distribution 10^5 ^times. All of the 7 samples yielded a goodness-of-fit p-value of greater than 0.5, suggesting that measured *HD*_0 _statistically follows a Poisson distribution. In this goodness of fit test the null hypothesis was that the two distributions tested were statistically the same, hence a low p-value would yield rejection of the null hypothesis. Analysis of all the sequence samples showed that the actual number of days elapsed following infection for the sequence samples fell within the 95% CIs of estimated days post infection by a Poisson fit to the *HD*_0 _distribution (Table 1). However, as we expected from the observed decrease in divergence and the increase in sequence identity as infection progresses, the correlation coefficient between actual days since infection and the estimated days post infection (based on the Poisson fit for animal r00065) was -0.91. The correlation coefficient for animal r98018 was 0.47.

## Discussion

The present study was undertaken to explore the applicability of a recently developed model for primary HIV-1 infection, to the analysis of acute SIV infection in rhesus macaques [2]. The level of measured diversity ranged from 0.015% to 0.052% during primary SIV infection, before set point, which is comparable to the range of measured diversity, 0.005% to 0.127%, from 68 single strain infected patients at the primary stage of HIV-1 infection [2]. Analysis of the SIV *nef *sequences showed that the MC simulation model was able to successfully classify 7 sequence samples, from two animals during the first 3 weeks following experimental infection of two rhesus macaques with SIVmac239, as homogeneous infection. We also confirmed that the consensus virus sequence in these animals was identical to the transmitted *nef *sequence of the infecting SIVmac239.

We observed an unexpected decline in the divergence and the diversity from animal r00065 at an early point following infection. We first hypothesized that the serial decline in the divergence might be due to fluctuations arising from the limited sample size, 31–50 sequences per time point. To address this concern, we performed a second simulation, starting with the actually sampled 41 nef genes obtained at day 7 from animal r00065 (which showed the divergence of 0.018%). The MC simulation was performed with the assumption of neutral evolution, and 31 sequences were sampled at day 18. The measured 95% CIs of the divergence from such 1000 simulations provided the basis for the rejection of the null hypothesis (neutral evolution without selection), implying a preferential selection process for the founder strain. We conclude that the decrease in the divergence observed in animal r00065 is reflective of a purifying selection rather than a stochastic effect due to small sample size. We speculate that the purifying selection can be explained as a result of either: (i) lower fitness of the emerging mutant viruses relative to the founder virus, or (ii) selective loss of mutant sequences due to linked, unfavorable changes elsewhere in the genome (i.e., the phenomenon of hitchhiking [29,30]). The roles of Nef in viral fitness, such as promoting viral replication and infectivity and interfering T cell activation, have been well documented [31-33].

**Figure 7:**
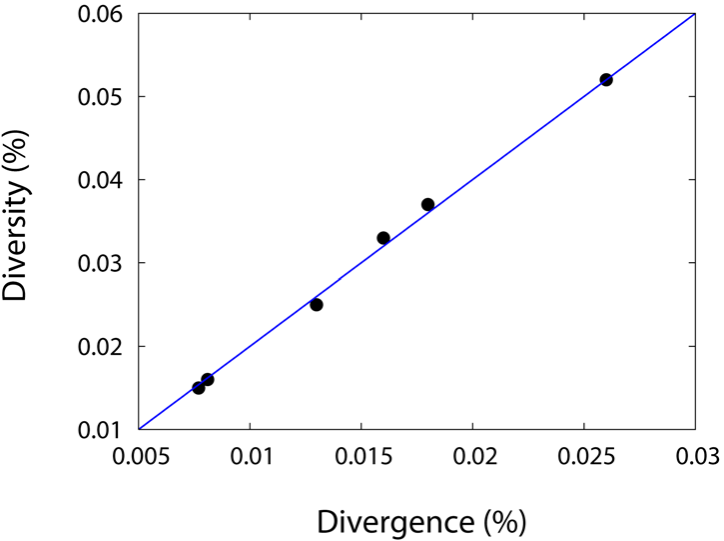
**Examination of star-like phylogeny**. The star-phylogeny can be examined by testing whether the level of diversity is two times of the level of divergence, which occurs when there is neutral selection in the absence of selective pressure for specific mutant strains. All of the 7 samples from animals r00065 and r98018 satisfy the relationship, diversity = 2 × divergence (blue line).

The time points in our study were chosen to precede the emergence of cytotoxic T cell lymphocyte (CTL) escape variants. As we expected, Figure 1 shows that all the mutants from the inoculated SIVmac239 *nef *gene are different each other, at the predicted amino acid level. This is not consistent with the expected outcome of CTL pressure, which classically results in changes confined within one or at most a handful of immunodominant epitopes. The main expected impact of CTL-induced changes on the model can be linked with a deviation from a star-like phylogeny [34], the absence of outgrowth in a particular mutant lineage. We have presented an examination of the property of star phylogeny in Figure 7 where all the 7 samples from two macaques satisfy the expected relationship for star-like phylogeny, diversity = 2 × divergence. The relationship arises from the property that the intersequence hamming distance frequency distribution coincides with the self-convolution of the frequency distribution of the hamming distances from the founder virus. The property of star-like phylogeny was preserved in all the samples from animal r00065 which displayed a sequential decrease in the divergence and the diversity (i.e., a purifying selection). Under the purifying selection preferential for the founder strain, a star-like phylogeny can be retained since there is no outgrowth in a particular mutant lineage except the center of the star, the founder virus.

We observed that rapid viral replication kinetics were not necessarily associated with a greater rate of sequence evolution. Animal r00065 displayed a greater level of viral replication in comparison to animal r98018 while less diversification of *nef *genes was observed in animal r00065. We interrogated the relationship between HIV-1 sequence diversity and viral load from 28 subjects with homogeneous HIV-1 infection in Fiebig stage II, where viral RNA and p24 antigens are positive without detectable HIV-1 serum antibodies [2]. We observed little correlation between plasma viral load and diversity (*σ*^2 ^= 0.18) in HIV-1 acute infection.

Disconnect between the replication rate and the rate of evolution during early SIV and HIV infections may be partly explained by the unusual small effective population size, which has been estimated ranging from 10^3 ^to 10^4 ^[35-38]. The effective population size is defined from the process of transforming an actual, census population into a neutral, constant size population with non-overlapping generations. The difference between the effective population size and the real size can arise from many factors such as varying population size, purifying or diversifying selection and the existence of subpopulation. These factors should be associated with low level of correlation between viral load and the level of diversity in acute HIV-1 and SIV infections.

Another aspect we may consider is that low level of correlation might be explained within our model scheme where the reproductive ratio and the generation time are set as independent parameters. Viral sequence diversity is influenced more strongly by generation time and to much lesser extent by the reproductive ratio. Hence for a given viral generation time, if the reproductive ratio changes significantly, the ramp-up slope of infected cell varies accordingly while the rate of sequence diversification remains relatively stable, implying little correlation between the rate of evolution and the rate of replication. For instance, our calculation from the asynchronous infection model study shows that when we change the basic reproductive ratio from 6 to 12, the ramp-up slope of infected cells increases 45% but the slope of diversity increases only 6%. With the assumption that the basic reproductive ratio varies considerably among acute HIV-1 subjects, for example by the level of activated CD4 T cell at the transmission, we may observe a great level of variation in the viral load but less in the sequence diversity. Under this circumstance, a minor correlation can be detected at the population level with another factor for dampening the correlation, fluctuations arising from the limited sample size of genes.

An important caveat to the work reported here is that a limited number of clones were examined at specific time points in only 2 SIV infected animals. SGA sequencing is resource-intensive, precluding the use of more animals and time points in this study. In the future, next-generation pyrosequencing technologies [39] may facilitate the examination of far greater numbers of SIV sequences with economy that is impossible to achieve with Sanger-based sequencing. We expect that the acute infection model will be refined and improved as additional sequences become available.

## Conclusion

This study verifies the robust nature of our MC simulation model for primary HIV-1 infection, and shows that it can be successfully applied to the analysis of acute SIV infection in rhesus macaques. The model predicted the level of SIV sequence diversification during the acute phase of SIVmac239 infection in two rhesus macaques, and it correctly identified "homogenous" virus transmission in this model system. SIV acute sequence samples confirmed that the consensus sequence of each sample was indeed the transmitted strain. Finally, a sequential decrease in viral diversity was observed during the first 3 weeks of infection in one macaque, and was found to be due to a purifying selection for the transmitted sequence.

## Methods

### Animals and SIVmac239 challenge

Two rhesus macaques were experimentally infected with the clonal SIV isolate SIVmac239, derived from a molecular clone [40]. The SIVmac239 inoculum was sequenced by non limiting dilution PCR. The sequence of the infecting strain was identical to the clone from which it was derived with potential small errors during in vitro amplification. We have indicated the limitation in the revised manuscript. However, we note that our method is the best way for obtaining the clonal nature of the infecting inoculum as far as we can. Animal r00065 (r65) was infected with 100 TCID_50 _SIVmac239 by intravenous injection. Animal r00098 (r98) was infected by intrarectal inoculation with 10 MID_50 _SIVmac239. Viral RNA was isolated from frozen plasma samples from animal r00065 collected at days 4, 7, 11, and 18 following virus infection. From animal r00098, viral RNA was isolated from frozen plasma samples collected at days 4, 7, 21 during infection. Virally-infected animals were cared for according to the regulations of the University of Wisconsin Institutional Animal Care and Use Committee, and the NIH.

### Viral RNA isolation and cDNA synthesis

Viral RNA was isolated from each animal at defined time points following infection. Cell-free plasma was prepared from EDTA anticoagulated whole blood by ficoll density gradient centrifugation. Viral RNA isolation was performed using the QIAamp MinElute Virus Spin Kit (QIAGEN, Valencia, CA) according to the manufacturer's instructions. Single strand cDNA was generated using oligo dT primers and the Superscript III reverse transcription kit (Invitrogen, Carlsbad, California, USA) according to the manufacturer's instructions.

### Limiting Dilution and nested PCR

cDNA template was diluted to ~1 viral genome per microliter. The dilution factor necessary to achieve single viral genomes was defined as the template dilution for which only 30% of reactions produced a product. According to a Poisson distribution, the cDNA dilution that yields PCR products in no more than 30% of wells contains one amplifiable cDNA template per positive PCR more than 80% of the time. This was empirically determined using a dilution series and varied between samples and cDNA preps. The dilution series and PCR reactions were set up using a QIAGEN BR3000 liquid handling robot (QIAGEN, Valencia, CA). All PCR reactions used Phusion High-Fidelity polymerase (Finnzymes, Espoo, Finland). A nested PCR approach was used for all amplifications. The following primers designed to amplify a region of the viral Nef gene were used for the first round of PCR: 5'-CAAAGAAGGAGACGGTGGAG-3' and 5'-CATCAAGAAAGTGGGCGTTC-3'. Second round PCR was conducted using 2 ul of the first round PCR product and the following internal primers were used for nested PCR: 5'-TCAGCAACTGCAGAACCTTG-3' and 5'-CGTAACATCCCCTTGTGGAA-3'. For all PCR reactions, the following conditions were used: 98C for 30 s, 30 cycles of: 98C for 5 s, 63C for 1 s and 72C for 10 s, followed by 72C for 5 min. PCR products were run on a 1.5% agaroe gel. PCR products were purified using the Chargeswitch kit (Invitrogen, Carlsbad, Calfornia, USA) according to the manufacturer's instructions. Samples were bi-directionally sequenced susing ET-terminator chemistry on an Applied Biosystems 3730 Sequencer (Applied Biosystems, Foster City, California, USA) and the internal primers described above. DNA sequence alignments were performed using CodonCode Aligner version 2.0 (CodonCode Corporation, Dedham, Massachusetts, USA).

### Modeling Sequence Evolution in Primary HIV-1/SIV Infection

The details of our model for characterizing sequence evolution in acute HIV-1 infection will be described by Lee et al. (HY Lee, EE Giorgi, BF Keele, B Gaschen, GS Athreya, JF Salazar-Gonzalez, KT Pham, PA Geopfert, JM Kilby, MS Saag, EL Delwart, MP Busch, BH Hahn, GM Shaw, BT Korber, T Bhattacharya, and AS Perelson, Modeling Sequence Evolution in Acute HIV-1 Infection, submitted for publication). We provide here an overview of the salient features of the model and its underlying assumptions. After transmission we assume that a systematic infection starts with a single infected cell in a new host. The number of secondary infections caused by one infected cell placed in a population of cells fully susceptible to infection is called the basic reproductive number, *R*_0_. The available data in humans infected with HIV-1 and in monkeys infected with SIV and SHIV show that virus grows exponentially until a viral load peak is attained a few weeks after infection [41-43]. Following the peak, viral levels decline and establish a set-point. At the set-point each infected cell, on average, successfully infects one other cell during its lifetime.

We assumed a homogeneous infection in which the virus grows exponentially with no selection pressure, no recombination, and a constant mutation rate across positions and across lineages. Cell infections occur randomly by the viruses released from an infected cell. Viral production starts on average about 24 hours after a cell is initially infected [44,45], and most likely continues until cell death. While each of the *R*_0 _infections could occur at different times, we took a first step in assessing the role of asynchrony by assuming the infections occur at two different times. The average time to new infection defines the viral generation time, *τ*. Each new infection entails a single round of reverse transcription introducing errors in the proviral DNAs with the number of mutations given by the Binomial distribution, *Binom*(*n*; *N*_*B*_, *ε*), where *n *is the number of new base substitutions. Binomial distribution implies that base substitutions occur independently with the probability of *ε *at each site of SIV genome with the length *N*_*B *_in each reverse transcription cycle. The Monte-Carlo model explicitly emulates all the new infection procedures with mutations, tracking the population of proviral *nef *genes of the infected cells by introducing base substitutions as infection propagates in a new host.

In Ref. [2], we determined that the MC simulation and the mathematical model showed a good agreement with the level of sequence diversity sampled from acute HIV-1 subjects presumably infected with a single variant. Based on the prediction made by the model, the group of identical sequences, usually the consensus sequence of sampled strains, was presumed to be the initial founder strain established by the systematic infection in each host. The parameters used in the acute HIV-1 model were: i) the average generation time of productively infected cells, defined as the average time interval between the infection of a target cell and the subsequent infection of new cells by progeny virions, estimated as 2 days [44], ii) HIV-1 single cycle forward mutation rate, estimated as *ε *= 2.16 × 10^-5 ^per site per cycle [46], and iii) the basic reproductive ratio, defined as the number of newly infected cells that arise from any one infected cell when almost all cells are uninfected, estimated as *R*_0 _= 6[41]. In the asynchronous infection model, the first time at which a newly infected cell infects other cells, *τ*, is chosen as 1.5 days. The length of *nef *gene, *N*_*B*_, we simulated is 792. We used these parameter values to analyze our data set. For example, calculated *R*_0 _values during primary SIV infection from viral ramp-up slope ranged from 2.2 to 68 [43], which justifies the choice of *R*_0 _= 6. Improvement of the model requires more accurate estimations for these basic parameters during SIV early infection.

The mutation rate, *ε*, and the generation time, *τ*, control the rate of increase in divergence and hence diversity. The larger the mutation rate, the faster the genomes mutate, hence the steeper the growth in diversity. The greater the generation time, the slower the genomes diversify, hence the smaller the growth in diversity. The slope of diversification is approximately proportional to *ε*/*τ*. On the other hand, *R*_0 _mainly controls the growth in the infected cell population size. As the viral population grows, the number of cells one infected cell infects decreases due to the fact that fewer cells are available for infection. The basic reproductive ratio, *R*_0_, affects the rate of evolution in a relatively minor way. Low values (e.g. 2 ≤ *R*_0 _≤ 4), slow down the growth in the infected cell population, thus affecting the speed of evolution. For example, from *R*_0 _= *6 *to *R*_0 _= *2 *there is a 15.9% increase in the slope of diversity. On the other hand, for *R*_0 _≥ 6, the dependence of the rate of diversification on *R*_0_is reduced. The slope of diversity increases by 5.5% as we increase *R*_0 _from 6 to 10. The dynamics of diversity do not depend on the number of initial infected cells.

Once we sample a finite number of sequences from the MC simulation at a given time, we first measure the Hamming distance (*HD*_0_) between each sampled sequence and the founder sequence and the Hamming distance (HD) between sequences sampled at the same time. Here Hamming distance is the number of base substitutions between two sequences. Based on the calculated *HD*_0 _and *HD*, we define the basic measurements for quantifying the evolution of HIV-1 sequence populations. Divergence is defined as the average *HD*_0 _per base from the initial founder strain; diversity is defined as the average intersequence Hamming distance per base among sequence pairs at a given time; variance is defined as the variance of the intersequence per base HD distribution; maximum HD is defined as the measured maximum HD between all sequence pairs sampled, and sequence identity is defined as the proportion of sequences identical to the founder strain. Both the MC simulation and mathematical calculation showed that divergence, diversity, and variance increase linearly as a function of time and sequence identity decays exponentially as a function of time [Fig. 2]. These behaviours are characteristics of neutral evolution, characterized as Poisson distribution and star-phylogeny topology. It has been shown that the distribution of pairwise genetic distances is an approximate Poisson in the evolution of mitochondrial DNA [28]. To address the issue of the finite size of samples, we repeated MC simulations sampling a finite number of nef genes at a given time and computed 95% CIs for each quantity. Then we examined whether the measurement of SIV *nef *gene samples was compatible with the model prediction or not. To infer the number of days elapsed since infection based on sampled strains, first we fit the Poisson distribution to the observed distribution of Hamming distances between sampled *nef *genes and the transmitted *nef *gene; we then determined the mean of the Poisson distribution and calculated days post infection using Eq. (2).

A key property of the Poisson distribution arising from neutral evolution without selection and recombination is that the level of diversity is comparable to that of variance. We used this property to examine whether sampled strains had evolved from a single founder strain or not. In each MC run, we obtained the values of diversity and variance from the sampled sequences with a given sample size at each time and located those values in the plane of diversity and variance. By repeating MC simulations, we collected all the values of diversity and variance and computed 95% CIs in the plane of diversity and variance. The computed 95% CIs form a conical region within which diversity and variance of the sampled sequences from the animal with homogeneous infection (i.e. infections with a single founder strain without any selection pressure or recombination) are expected to be located [Figure 5]. As we sample more, the conical region becomes smaller [Figure 5]. Another requirement for homogeneous infection is that the sequence diversity should be less than the upper limit of the 95% CIs of the diversity at a given time following infection with a single virus strain.

## Competing interests

The authors declare that they have no competing interests.

## Authors' contributions

BNB and DHO performed the animal experiment and *nef *gene SGA sequencing. HL performed the sequence data analysis and model simulations. EEG and ALA were responsible for the statistical analysis including the Poisson fit. PC, BK, SD, and HL were responsible for design and writing of the manuscript. All authors read and approved the final manuscript.
